# Exploring Responses to Body Weight Criticism: Defensive Avoidance When Weight Is Seen as Controllable

**DOI:** 10.3389/fpsyg.2020.598109

**Published:** 2020-12-07

**Authors:** Susanne Täuber, Stuart W. Flint, Nicolay Gausel

**Affiliations:** ^1^Department of Human Resource Management and Organizational Behavior, University of Groningen, Groningen, Netherlands; ^2^School of Psychology, University of Leeds, Leeds, United Kingdom; ^3^Scaled Insights, Nexus, University of Leeds, Leeds, United Kingdom; ^4^Faculty of Health and Welfare, Østfold University College, Fredrikstad, Norway

**Keywords:** weight moralization, obesity controllability beliefs, body mass index, self-to-self relating, defensive avoidance, belongingness and acceptance

## Abstract

In Western society, weight moralization is reflected in the belief that weight is controllable across the weight spectrum. However, the effect of holding such beliefs is unclear. We therefore propose that these beliefs affect people differently depending on their BMI. When confronted with negative, self-related feedback, people’s coping strategies are often reflected in the ways they relate to their self. We examine three such self-to-self relations (i.e., reassured, inadequate, and hated self). Extending prior research, we predict that weight controllability beliefs are related to positive self-to-self relations for adults with a low, and to negative self-to-self relations for adults with a high BMI. Accordingly, we expected that weight controllability beliefs would be associated with defensive avoidance among people with a high, but not with a low BMI. We tested our hypotheses in a sample of 348 adults who participated in an online survey. Weight controllability beliefs were associated with increased defensive avoidance in people with high BMI, and with decreased defensive avoidance in adults with a low BMI. Forms of self-to-self relating fully mediated this association, demonstrating positive effects on adults with a low, and negative effects on adults with a high BMI. Additionally, in an open ending section, we found seven social settings that deprive people from satisfying their need to belong and to be accepted due to their weight. We discuss our findings against a call for a less moralized public discourse about overweight and obesity that is particularly relevant in the context of the current COVID-19 pandemic.

## Introduction

Body weight is a highly moralized issue in public discourse and healthcare (Rozin, 1999; Townend, 2009; Flint et al., 2016; Täuber, 2018). The societal norm that a thin body weight or one in the “normal” range is healthy and under one’s control unfairly portrays people living with overweight and obesity as a living, walking illustration of a moral failure. A core element of moralization is that the desired personal characteristic—in this case, a body weight that conforms to society’s standards—is perceived as controllable, and thus reflects the moral quality of effort (Weiner, 2001; Täuber, 2018), and self-control (Salthe and Gausel, 2014). The societal belief that weight is controllable, and overweight thus reflects a lack of morality through failure of effort and self-control, is shared not only by the wider population, but has also been internalized by people living with obesity (Duarte et al., 2014; Täuber et al., 2018)^1^.

In modern Western societies, a thin body shape stands synonymous for moral traits such as self-discipline and willpower (Crandall, 1994; Crandall et al., 2001; Duarte et al., 2015). As such, society, and indeed many people who internalize societal views of weight statuses, perceive thin, athletic weight statuses as a desirable, and overweight as an undesirable personal characteristic. In their role as “naïve scientists” (Heider, 1958), people typically try to understand the causes of undesirable personal characteristics, to determine whether punishment or pity is the most appropriate reaction. Such judgments follow a specific hierarchical pattern in which observers of negative personal characteristics assign controllability, responsibility, and blame. These hierarchical patterns are condensed in decision stage models of attribution (Mantler et al., 2003), which emphasize the implications of controllability attributions on ascriptions of responsibility and consequently blameworthiness for a personal characteristic. Indeed, substantial evidence demonstrates that attributions of personal responsibility influence weight stigma and weight-based discrimination because they lead to blame toward people living with obesity (e.g., Puhl and Luedicke, 2012; Flint et al., 2015; Ringel and Ditto, 2019; Täuber, 2019).

Supporting the notion of moral failure, stereotypes of people with obesity often revolve around their supposed lack of moral integrity; that they lack willpower, that they are lazy, and gluttonous (Puhl and Brownell, 2006; Täuber et al., 2018). As it is deeply unpleasant to face harsh, stigmatizing accusations from others (Ellemers and van den Bos, 2012; Gausel and Bourguignon, 2020), those in focus of the accusations typically try to psychologically defend against it by resorting to various defensive strategies (Gausel and Leach, 2011; Gausel, 2013; Täuber et al., 2018). Some defensive strategies which allow a person to participate in social situations while minimizing the risk of condemnation are the motivations to hide and cover-up the unwanted aspect of oneself (e.g., Gausel et al., 2012) or, in terms of body-image and obesity, to conceal the body (Duarte et al., 2015). However, if the anticipated risk of condemnation is judged to be too high, it is likely that people will defensively avoid social situations altogether (Gausel, 2013). That is, they will physically avoid social situations, or people, where condemnation can be most likely anticipated (Gausel et al., 2016) such as going to places where the body will be in focus (Duarte et al., 2015). Hence, defensive avoidance reflects the motivation to minimize or even hinder exposure of a person’s body so one can escape anticipated, forthcoming weight-based condemnation.

Defensive avoidance should be closely related to how people think about themselves when confronted with failure, criticism, or distress. In the coping literature, self-criticism is perceived as a maladaptive way of relating to the self (Gilbert et al., 2004) and has been associated with depression, anxiety, and also eating disorders (cf., Sommers-Spijkerman et al., 2018). Self-assurance, on the other hand, is perceived as an adaptive form of relating to the self that contributes to mental health and wellbeing and protects against psychological distress such as that arising from weight stigma. As such, health psychologists have suggested that self-to-self relating forms a critical process in coping with, adapting to, and recovering from distress. Our current study tests the focal hypothesis that beliefs about weight controllability critically shape psychological responses to distress, operationalized through forms of self-to-self relating, but that they do so in dramatically different ways among people with and without overweight and obesity.

Specifically, based on the above considerations, we suggest that beliefs about weight controllability negatively affect people living with overweight and obesity, who will perceive their weight status as reflecting an undesirable personal characteristic, leading to self-blame. By contrast, we expect beliefs about weight controllability to positively affect people living without overweight and obesity, who will associate their weight status with a desirable personal characteristic, leading to self-praise. We propose that these appraisals of the self will be reflected in three forms of “self-to-self relations”; the reassured self, the inadequate self, and the hated self. The ability to reassure oneself is considered an adaptive form of self-to-self relating, reflecting a “positive, warm and accepting attitude toward the self” (Sommers-Spijkerman et al., 2018, p. 730). The inadequate and hated self are both considered maladaptive forms of self-to-self relating. While both involve self-criticism, the inadequate self reflects a desire to correct or improve those aspects of the self that are criticized. By contrast, the hated self reflects a desire to “hurt, persecute and attack the self” (Sommers-Spijkerman et al., 2018, p. 730). Importantly, the inadequate and hated selves have been linked to various negative outcomes such as depression, anxiety, eating disorders, and self-injury (c.f., Sommers-Spijkerman et al., 2018).

## The Current Study

Whilst evidence has accumulated about the stigmatizing attitudes toward people living with obesity and the experiences of weight stigma and discrimination (e.g., Ringel and Ditto, 2019; Hunger et al., 2020; Pudney et al., 2020), less is known about how it relates to motivations to psychologically defend against anticipated condemnation of one’s body-image (i.e., part of one’s body or the whole body, but see Gausel and Leach, 2011). Hence, our study examined whether beliefs about the controllability of obesity will exert different effects based on weight status in relation to the self-concept, and how beliefs about the controllability of obesity would predict defensive avoidance (of relevant contexts and concealment of the body). Finally, our study explored and identified different settings that people typically avoid in relation to their weight status. We believe our study can aid healthcare professionals and professional organizations to support people with body-image concerns and obesity in their understanding of problems related to the self and how people try to manage and defend against possible condemnation from others. Our findings further tie in with scholarly calls for a less moralized public discourse about weight (e.g., Townend, 2009; Flint, 2015; Rubino et al., 2020).

**FIGURE 1:**
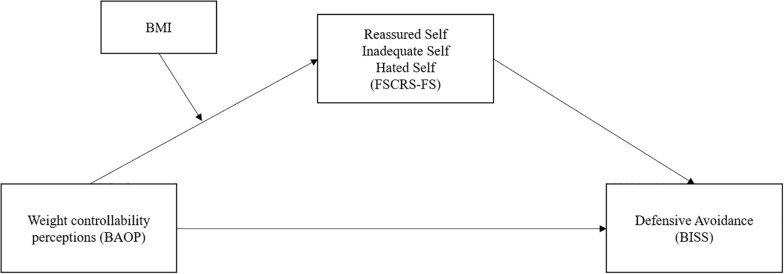
The predicted moderated mediation model, testing the interactive effect of Weight Controllability Beliefs and BMI on Defensive Avoidance through Self-to-self-relating.

Previous research has demonstrated that weight controllability beliefs are high across the weight spectrum (e.g., Crandall and Martinez, 1996). Going beyond prior research, the current study examined whether the effects of these beliefs will differ based on weight status. Specifically, we predicted that weight controllability beliefs are associated with negative forms of self-to-self-relating in people with a high, but with positive forms of self-to-self-relating in people a low BMI (*Hypothesis 1*). We further predicted that the interactive effect of weight controllability beliefs and BMI on defensive avoidance is mediated by self-to-self-relating (*Hypothesis 2*). Figure 1 depicts the complete moderated mediation model that we test. In addition to these hypotheses, we aimed to explore the settings where people typically report avoidance in relation to their body shape.

To test our hypotheses, we used the same dataset as reported in Täuber et al. (2018). The focal outcome of the complete research project was defensiveness. In the model proposed and tested in Täuber et al. (2018), defensiveness was conceptualized as self-determined vs. other-determined regulation of dieting and exercising and was predicted by people’s perception of what others thought of them. Thus, defensiveness was modeled as a consequence of *people’s relationships with others*. The current study is complementary to in Täuber et al. (2018). Here, we conceptualize defensiveness as defensive avoidance (a more direct measure of defensiveness compared to self-regulated vs. other-regulated motivation) and model it as a resulting from *people’s relationship with the self*. We predict that this relationship with the self is affected by people’s individual weight controllability beliefs, but that the effect varies as a function of the person’s own weight, as indicated by their BMI.

## Methods

### Participants and Procedure

Participants were invited to take part in the study through *MTurk* participant pooling^2^. Prior to participating in the study, all participants read the standard online study information sheet and provided informed consent by clicking “yes” or “no” in response to the statement “I consent.” Only participants clicking “yes” were then presented with the survey questions. Participants clicking “no” were presented with a page thanking them for their time and finishing their participation. Three-hundred-fifty-one U.S. American respondents were recruited (respondents received $2 as compensation), reflecting a sample size that is common for survey research at the authors’ institutions. Three participants were excluded due physically implausible BMI values resulting from their reported weight and height (0.19, 3.87, and 11.08 kg/m^2^, respectively). The resulting sample of 348 respondents consisted of 181 females (52%) and 167 males (48%), *M_*Age*_* = 37.15, *SD_*Age*_* = 11.15, *M_*BMI*_* = 26.78, *SD_*BMI*_* = 6.78, range 15.34–65.10^1^. The recruited sample demonstrated a diverse body weight that allowed for testing the hypotheses. Data checks were performed to ensure the quality of the data^3^.

### Measures

The Beliefs About Obese Persons scale (Allison et al., 1991) was used to assess respondents’ *beliefs about the controllability of obesity* (α = 0.74), where higher values indicate stronger beliefs that obesity is controllable. To assess the three forms of self-relating; *self-reassurance* (α = 0.91), *self-inadequacy* (α = 0.87), and *self-hatred* (α = 0.85), the Forms of Self-Criticizing/Attacking and Self-Reassuring Scale Short Form (Sommers-Spijkerman et al., 2018) was used. As we were interested in the behavioral motivation of how people react to anticipated criticism of the self, we were inspired by other researchers’ (Gausel et al., 2012, 2016; Duarte et al., 2015) focus on people’s motivation to psychologically defend their self with physical and social avoidance and wanting to cover-up and hide. As our study specifically focuses on obesity and the presentation of body-image, we deployed the measures originally developed by Duarte et al. (2015) to contextualize the motivation to resort to *defensive avoidance* (α = 0.97). Finally, we asked respondents to freely indicate settings they tended to avoid in an open question. All three authors coded the open-ended responses independently. Disagreements were resolved through discussion and reflection. In the Supplementary Appendix, we provide an overview of the complete questionnaire, including all scales and associated items.

### Statistical Analyses

We tested the hypotheses in a stepwise approach. First, we ran correlational analyses to provide an overview of the associations between variables. Second, we tested the predicted moderated mediation model as depicted in Figure 1 using the PROCESS macro (Hayes, 2012; model 7; 5,000 bootstrap intervals, predefined). In our model, weight controllability beliefs were the independent variable, the three forms of relating to the self were parallel mediators, and defensive avoidance was the dependent variable. BMI was modeled as a continuous moderator of the association between the independent variable and the mediators. All variables were z-standardized. All constructs were continuous and z-standardized.

## Results

### Descriptive Analyses and Weight Controllability Perceptions

Respondents’ height and weight were used to calculate body mass index (BMI). Our sample was comprised of 15 respondents in the underweight range (< 18.5 kg/m^2^), 149 in the normal weight range (18.5–24.9 kg/m^2^), 100 in the overweight range (25–29.9 kg/m^2^) and 84 in the obesity range (≥ 30 kg/m^2^). Table 1 gives an overview over correlations between sex (female = 1, male = 2), age, and BMI with weight controllability beliefs, defensive avoidance and the three forms of self-to-self relating. Interestingly, controllability beliefs showed no significant association with other variables. By contrast, strong associations in the expected directions were found between defensive avoidance and forms of self-to-self relating. Specifically, reassured self was negatively associated with BMI and with defensive avoidance, while inadequate and hated self were positively associated with BMI and with defensive avoidance. Respondents’ beliefs that weight is controllable was significantly above the midpoint of the scale (*M* = 5.17, *SD* = 0.08), *t*(347) = 27.26, *p* < 0.001. In alignment with prior research, this pattern did not co-vary with respondents’ BMI (*r* = −0.06, *p* = 0.24).

**TABLE 1:** Means, standard deviations, and correlations between demographic variables, BMI, beliefs about the controllability of obesity, defensive avoidance, and views of the self as reassured, inadequate, and hated (Forms of Self-Criticizing/Attacking and Self-Reassuring Scale).

	*M*	*SD*	1	2	3	4	5	6	7	8
1. Sex^*a*^	1.48	0.50	1.00							
2. Age	37.15	11.15	−0.14*	1.00						
3. BMI	26.78	6.78	−0.01	0.00	1.00					
4. Beliefs about controllability of obesity	5.17	0.80	−0.06	−0.09	−0.06	1.00				
5. Defensive avoidance	2.41	1.13	−0.25***	−0.04	0.43***	0.04	1.00			
6. Reassured self	3.48	1.03	0.01	0.05	−0.16**	−0.02	−0.54***	1.00		
7. Inadequate self	2.39	1.39	−0.07	−0.18**	0.22***	0.08	0.67***	−0.59***	1.00	
8. Hated self	1.74	0.92	−0.03	−0.22***	0.26***	0.03	0.65***	−0.52***	0.77***	1.00

*p < 0.05. **p < 0.01. ***p < 0.001. ^*a*^1 = female, 2 = male.

### Moderated Mediation Hypothesis

Because of the associations found between demographic variables and some of the focal constructs (Table 1), we controlled for age and sex in the analysis^4^. Conventionally, BMI is considered low when smaller than 18.5, normal when between 18.5 and 25, and high when exceeding 25. In our particular sample, BMI was 21.14 at one standard deviation below the mean (corresponding to the low-mid point of conventional “healthy weight” BMI), 25.11 at the mean (corresponding to just exceeding the boundary between “normal” and “overweight” BMI according to conventional cut offs), and 32.49 at one standard deviation above the mean (corresponding to “overweight” and “obese” BMI according to conventional cut offs). In the z-transformed metric, these values correspond to −0.83, −0.25, and 0.84, respectively. Table 2 provides an overview over the findings.

**TABLE 2:** Mediator model and dependent variable models testing moderated mediation (model 7, PROCESS, Hayes, 2012).

Predictor	B	SE	*t*	*p*	CI_95__%_
**Mediator variable model reassured self (*R*^2^ = 5.21)**					
Constant	−0.01	0.26	−0.20	0.844	−0.114, 0.094
Weight controllability beliefs	−0.03	0.05	−0.64	0.526	−0.139, 0.071
BMI	−0.17	0.05	−3.19	0.002	−0.273, −0.065
Interaction	−0.16	0.06	−2.81	0.005	−0.280, −0.049
Age	0.06	0.05	1.10	0.271	−0.004, 0.164
Sex	0.02	0.05	0.41	0.683	−0.083, 0.127
**Mediator variable model inadequate self (*R*^2^ = 13.31)**					
Constant	0.01	0.05	0.27	0.785	−0.086, 0.113
Weight controllability beliefs	0.10	0.05	1.92	0.056	−0.003, 0.198
BMI	0.24	0.05	4.66	< 0.001	0.136, 0.336
Interaction	0.21	0.06	3.74	< 0.001	0.099, 0.319
Age	−0.19	0.05	−3.71	< 0.001	−0.290, −0.089
Sex	−0.10	0.05	−1.96	0.050	−0.201, 0.000
**Mediator variable model hated self (*R*^2^ = 16.57)**					
Constant	0.02	0.05	0.32	0.748	−0.082, 0.114
Weight controllability beliefs	0.04	0.05	0.79	0.427	−0.059, 0.138
BMI	0.27	0.05	5.39	< 0.001	0.170, 0.365
Interaction	0.24	0.05	4.42	< 0.001	0.135, 0.351
Age	−0.23	0.05	−4.61	< 0.001	−0.330, −0.133
Sex	−0.05	0.05	−1.09	0.275	−0.153, 0.044
**DV model: defensive avoidance (*R*^2^ = 56.77)**					
Constant	0.00	0.04	0.02	0.981	−0.069, 0.071
Self-assurance	−0.20	0.04	−4.56	< 0.001	−0.292, −0.116
Self-inadequacy	0.29	0.06	4.76	< 0.001	0.170, 0.409
Self-hatred	0.33	0.06	5.72	< 0.001	0.216, 0.443
Weight controllability beliefs	0.02	0.04	0.63	0.532	−0.048, 0.094
Age	0.07	0.04	0.95	0.052	−0.001, 0.146
Sex	−0.21	0.04	−5.71	< 0.001	−0.279, −0.136

*The interaction term refers to the interaction between weight controllability beliefs and BMI.*

**FIGURE 2:**
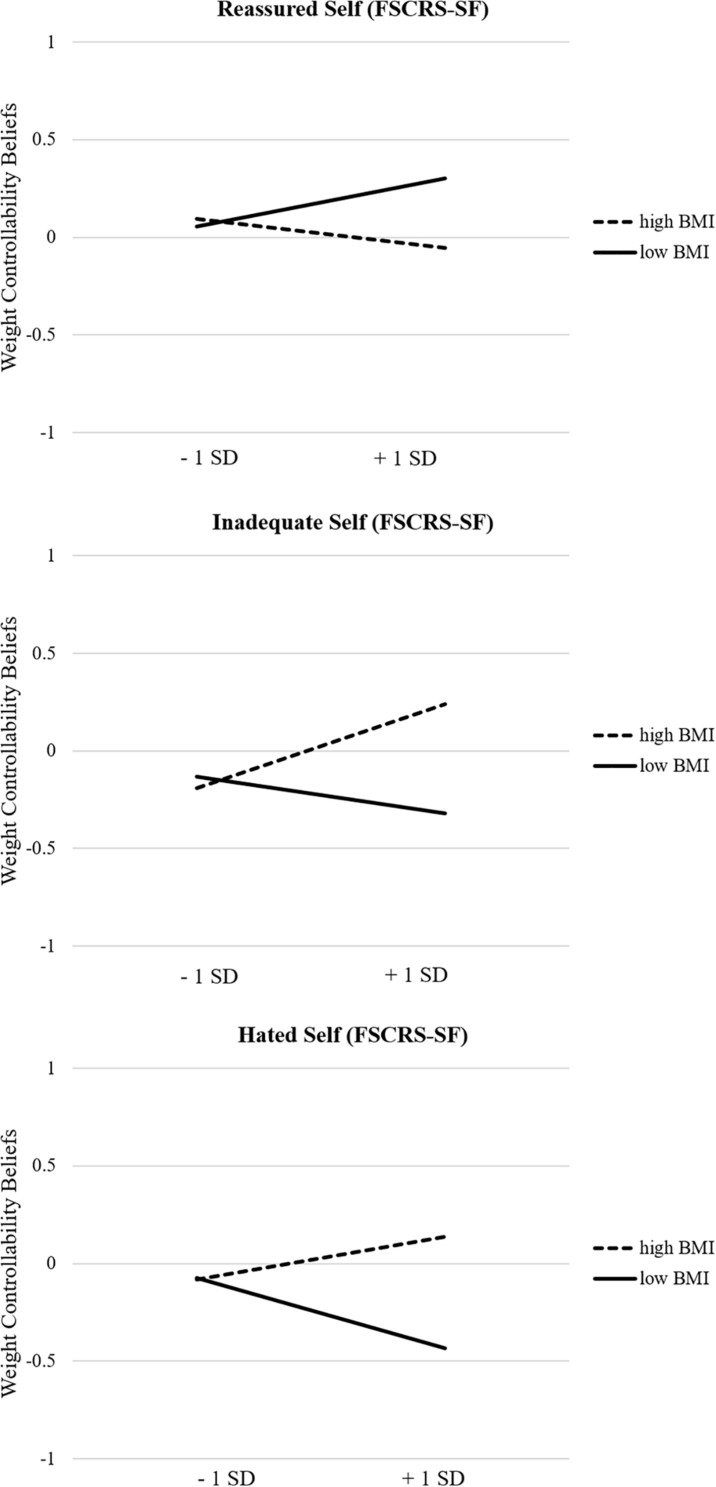
**(Upper panel)** Simple slopes for the effect of weight controllability beliefs on self-reassurance for respondents with low and high BMI. (Middle Panel). Simple slopes for the effect of weight controllability beliefs on self-inadequacy for respondents with low and high BMI. **(Lower panel)** Simple slopes for the effect of weight controllability beliefs on self-hatred for respondents with low and high BMI.

Interpreting the moderations first, reveals that the interaction terms were significant for all self-to-self relations. Simple slope analyses revealed that weight controllability beliefs were associated with significantly lower self-reassurance in people with a high BMI (β = −0.20, *t* = −2.43, *p* = 0.015), whilst the slope for people with a low BMI was non-significant (β = 0.13, *t* = 1.65, *p* = 0.101; see Figure 2, upper panel). Furthermore, weight controllability beliefs were associated with significantly higher self-inadequacy in people with a high BMI (β = 0.31, *t* = 3.86, *p* < 0.001), whilst the slope for people with a low BMI was non-significant (β = −0.09, *t* = −1.28, *p* = 0.203; see Figure 2, middle panel). Finally, weight controllability beliefs were associated with significantly higher self-hatred among people with a high BMI (β = 0.29, *t* = 3.95, *p* < 0.001). The slope for people with a low BMI was also significant (β = −0.18, *t* = −2.43, *p* = 0.015), indicating that weight controllability beliefs were associated with significantly lower self-hatred in people with a low BMI (see Figure 2, lower panel).

The mediator models show that greater self-reassurance was associated with lower motivation to defensively avoid social situations among respondents, while greater perceptions of the self as inadequate and hated were associated with greater motivation to defensively avoid social situations among respondents. Testing whether the meditations of weight controllability beliefs on defensive avoidance was indeed moderated by respondents’ BMI, the conditional effects show that self-reassurance mediated the association between beliefs about the controllability of obesity and defensive avoidance at high levels of BMI (+ 1 SD, *CI_95__%_* 0.007, 0.099). Self-inadequacy mediated the association between beliefs about the controllability of obesity and defensive avoidance at average and high levels of BMI (0 and + 1 SD; *CI_95__%_* 0.001, 0.065 and *CI_95__%_* 0.041, 0.158, respectively). Finally, self-hate mediated the association between beliefs about the controllability of obesity and avoidance at low and high levels of BMI (− 1 SD and + 1 SD; *CI_95__%_* −0.119, −0.030, and *CI_95__%_* 0.037, 0.165, respectively). Providing support for *Hypotheses 1* and *2*, forms of relating to the self mediated the effect of weight controllability beliefs on defensive avoidance, an effect that was moderated by BMI (index of moderated mediation *CI_95__%_* 0.010, 0.075, *CI_95__%_* 0.028, 0.106, and *CI_95__%_* 0.043, 0.128 for self-reassurance, self-inadequacy, and self-hatred, respectively).

### Settings That Respondents Avoid

Ninety-six respondents (27.6%) used the open question to describe which settings they tended to avoid. We checked for differences between respondents who did vs. did not answer the open question. A *t*-test revealed that respondents answering the open question had a significantly higher BMI (*M* = 29.33, *SD* = 7.96) compared to those not answering the open question (*M* = 25.81, *SD* = 6.01), *t*(346) = 4.45, *p* < 0.001. In addition, respondents answering the open question in general scored significantly higher on defensive avoidance in relation to their body-image (*M* = 3.17, *SD* = 1.02) than those not answering the open question (*M* = 2.12, *SD* = 1.03), *t*(346) = 8.54, *p* < 0.001).

We identified seven categories through coding the answers independently, and solving disagreement through discussion. During the coding process, we grouped together social settings that respondents’ perceived as exposing their bodies, prompting condemnation, ridicule, and judgment by others, and triggering feelings of shame and uneasiness. The setting most avoided (62 counts, 59.25%) were the ones involving social situations where it is expected to expose larger parts of the body, such as going to swimming pools and beaches. As an illustration, one respondent wrote: “*I don’t go swimming, but I love swimming.*” The second-most reported setting (19 counts, 18.24%) involved social situations where there is an expectation to wear clothes that underline body shape, such as going out to nightclubs, parties, or weddings. For instance, one respondent stated: “*I refuse to go downtown with my friends, even on my 21st birthday.*” The third-most reported setting (15 counts, 14.4%) were the ones involving a body-focus such as going to the gym, spa, and other fitness centers. To illustrate, one respondent wrote: “*I do not go to gyms as you always get judged.*” The fourth-most reported setting (11 counts, 10.56%) related to social situations involving food, such as grocery shopping or going to restaurants. One respondent wrote: “*I hate grocery shopping because I feel like I’m being judged by what is in my shopping cart.*” The fifth-most reported setting, and mentioned as often as the previous one (11 counts, 10.56%) involved situations where one could encounter people in clothes stores. As an illustration, one respondent wrote: “*I avoid shopping for clothing in thin people stores when there are a lot of people in the store.*” The sixth-most reported setting (9 counts, 8.64%) involved situations where one would be placed in front of others such as presenting a piece of work in front of others. Illustrating this, one respondent wrote: “*I do not volunteer to present at work because I don’t want to be seen by my coworkers.*” The seventh setting (2 counts, 1.92%), reported was avoiding health-related situations where one could be accused of being at fault for one’s own medical condition, such as going to the medical doctor’s office. As an illustration, one respondent wrote: “*I think that maybe the doctor and nurses think that I am just a glutton when in reality I don’t eat very much at all.*”

## Discussion

Against the background of the highly moralized discourse regarding obesity in Western societies (Rozin, 1999; Townend, 2009; Flint et al., 2016; Täuber, 2018), we predicted that weight controllability beliefs would negatively affect people with overweight and living with obesity, contrary to people without overweight and obesity. Our findings support this reasoning: beliefs about weight controllability were associated with higher levels of defensive avoidance among adults with high BMI, an effect that was mediated by less positive and more negative self-to-self relations. By contrast, beliefs about weight controllability were associated with lower levels of defensive avoidance among adults with low and normal BMI, an effect that was mediated by more positive and less negative self-to-self relations. We thus extend and complement prior research by showing the relevance of beliefs about weight controllability for self-to-self relations (Gilbert et al., 2004; Sommers-Spijkerman et al., 2018), and defensive avoidance (Weiner, 1985; Duarte et al., 2015).

Even though we did not investigate maladaptive consequences of being judged and condemned, other studies have found that depression, anxiety, eating disorders, and self-injury are related with body image concerns and overly self-critical forms of relating to the self (Gilbert et al., 2004; Duarte et al., 2015; Sommers-Spijkerman et al., 2018). Thus, beliefs about weight controllability can be viewed as an important determinant of how people relate to others and to the self. Indeed, while research has established that weight-stigma and weight-based discrimination result from beliefs about weight controllability (e.g., Weiner et al., 1988; Crandall and Martinez, 1996; Weiner, 2001; Mantler et al., 2003), our research advances existing insights by showing that similar processes unfold when people with overweight and obesity judge themselves in response to their feared concern for moral condemnation (see Gausel and Leach, 2011).

In response to body weight and body-image, we asked our participants to respond to the open-ended section of the questionnaire in order to specify which, if any, settings they tended to avoid. As expected, those who answered these open-ended questions had a higher BMI than those who did not respond, given the generally stronger desire to defensively avoid among respondents with a high, as compared to a low, BMI. This finding supports Duarte et al. (2015) and Täuber et al. (2018) argument that it is those who are in the focus of body-weight accusations that tend to avoid settings with a body-focus. It also supports Gausel and Leach’s (2011) and Gausel (2013) theorizing on psychological defense that it is those who carry an unwanted aspect of the self that most need to psychologically and socially defend against it.

The answers to the open-ended section provided us with seven categories of settings that tended to be avoided. Even though they all involve an expectation of body exposure, or carry a body-focus and food/health focus, we would like to highlight the motivation to avoid places of social gathering (such as swimming pools, beaches, parties, and weddings). That these settings are avoided is understandable as there’s either a strong expectation that parts of the body should be exposed or that clothes should be worn that accentuate body shape. Understandably, the way we socially self-represent is associated with our need to belong and feel emotionally accepted (Pardede et al., unpublished). However, avoiding these social settings will typically deprive the person with overweight or obesity from being with friends and family, and will thus hurt their feeling of belongingness (Baumeister and Leary, 1995) and acceptance (Hayes, 1994). Undoubtedly, when “everyone else” is being together on sunny summer days or in social settings, to be deprived of these experiences due to concerns about their weight and body shape can result in deeply unpleasant feelings of isolation and rejection (Gausel and Leach, 2011). Another finding that we regard as a serious concern, was respondents’ reported avoidance of seeking medical help, such as going to the doctors’ office. We elaborate on the implications of such avoidance in greater detail below, where we consider the relevance of our research in the context of the ongoing coronavirus (COVID-19) pandemic.

Our findings offer a strong pointer toward the negative consequences of weight moralization for those targeted in policy, health, and media campaigns: Through increasing beliefs that obesity is controllable, moralized discourse harms people living with obesity. Rather, policy health and media campaigns should provide empathy and compassion, with a focus on supporting people to engage in healthy behaviors. This underscores the need for morally neutral discourse concerning overweight and obesity. Given the severely negative consequences of moral condemnation (Gausel and Leach, 2011; Täuber, 2018, 2019), judgment of people with overweight and obesity and the associated overly critical forms of relating to the self (Duarte et al., 2015; Sommers-Spijkerman et al., 2018), our research suggests that interventions need to focus on two groups of people differently: On the one hand, interventions should aim to decrease weight stigma and discrimination in people across the weight spectrum. On the other hand, interventions should aim to decrease perceived moral condemnation, the associated negative relations to the self, and the resulting maladaptive behavioral responses in people with overweight and obesity.

In line with the reasoning presented here, we suggest that one potent route to achieve this is by targeting people’s beliefs about the controllability of obesity, utilizing substantial existing evidence. For instance, the Foresight Report (Butland et al., 2007) informs that the causes of obesity are complex and multi-level with many contributing factors outside of a person’s control such as genetics or food and drink marketing. Greater efforts to disseminate information to the public that provides a more accurate picture, rather than the current discourse that is devoid of information about factors outside of an individual’s control (Flint et al., 2016, 2018), appears warranted. There is ample evidence demonstrating that higher beliefs that obesity is controllable and associated with more stigmatizing attitudes toward people with obesity (e.g., Flint et al., 2015). Where obesity is perceived to be controllable and thus, within personal responsibility, this leads to blame. Research has shown that education about the factors outside of an individual’s control that also influences weight status, and thus reduces personal responsibility, has been shown to reduce weight stigma attitudes (e.g., O’Brien et al., 2010; Diedrichs and Barlow, 2011). Given the association between experiences of weight stigma and discrimination and reduced healthcare seeking behavior, it might also be expected that reducing personal responsibility associated with obesity might also lead to increased health seeking behavior. Research that explores this potential effect is warranted. In this respect, we believe that an important task lies in closer collaboration between public and patients, scientists, healthcare professionals, and communication experts to develop campaigns that can walk this tightrope.

## Relevance of Our Findings in the Context of COVID-19

Apart from health campaigns, public discourse during the COVID-19 pandemic underlines the necessity of non-stigmatizing language regarding people with obesity. Our findings suggest at least three ways in which the pandemic will have particularly detrimental effects on people living with obesity: First, despite some evidence that questions the link between obesity and severe illness (Ioannou et al., 2020), research and public messaging has informed that there is an association between obesity and increased hospitalization and death from COVID-19 were reported early on (e.g., Lighter et al., 2020). To the extent that obesity is attributed to a moral failure of the people themselves, this can increase stigma (i.e., “she brought that disease on herself”), leading to bias decision making and access to support (e.g., Weiner et al., 1988; Mantler et al., 2003). These dynamics are particularly concerning when considering the increased demand on hospitals which makes triage decisions more likely. We consequently strongly advocate for ethic councils and other advisory bodies to governments during the pandemic to be aware of the real possibility that weight moralization leads to biased decisions against people with overweight and obesity. Second, experiences of weight stigma and discrimination and internalization of weight bias has been associated with both mental (e.g., increased depression, reduced self-esteem), and physical health (e.g., increased cardio-metabolic risk factors) health concerns.

Third, reflecting the internalization of controllability attributions regarding obesity, people living with obesity may be more inclined to avoid seeking specialist medical help, out of fear of being judged, ridiculed, or denied. Thus, while accumulating evidence informs of a greater risk of severe illness and death from COVID-19 (Lighter et al., 2020), people living with obesity may be hesitant to seek healthcare support. Indeed, our open answers provided evidence that people with overweight and obesity avoided seeking medical support for fear of being judged. Fourth, measures to contain the pandemic require large-scale social isolation. Social isolation is at the heart of weight stigma (Ryan et al., 2020); hence, people living with obesity may suffer from the associated mental strain more than non-stigmatized subgroups of the population. Indeed, recent research demonstrates that the experience of weight stigma is associated with an increased psychological impact of the COVID-19 response (e.g., Frühbeck et al., 2020; Le Brocq et al., 2020; Puhl et al., 2020).

## Limitations and Conclusion

First, we employed a cross-sectional survey method, which is not suited to make causal claims. Thus, in theory, it is possible that people with overweight and obesity who already have an overly critical relation to the self tend to believe that obesity is more controllable. Complementing the research reported here with experimental designs that manipulate the extent to which weight is depicted as controllable can address this issue. Second, we acknowledge that sample size determination should be guided by more sophisticated measures than following common practice. Conducting *a priori* power analyses (e.g., Faul et al., 2009) helps determine the required sample size, which allows to derive accurate and stable effect size estimates (Lakens and Evers, 2014). Thus, despite conceptually replicating prior research, our results reported here should be interpreted with caution, because we cannot determine *post hoc* whether the sample size was big enough to ensure stable and accurate effect size estimates (Ellis, 2010). Third, we did not ask for race and ethnicity demographics, and thus cannot control for how BMI varies across these demographics, nor how they affect self-to self-relating or our other variables. Future research should take these variables into account. Finally, there are limitations to using BMI to reflect weight status, with ample research indicating that people may under- or in some instances, over-report their BMI when self-reported rather than objectively measured (Bowman and DeLucia, 1992; Gorber et al., 2007). Because BMI is not a direct measure of body composition (body fat), a high BMI does not indicate obesity in a strict sense of the term, as an obese BMI doesn’t necessarily mean that there are health impairments. Future research should use combinations of subjective and objective measures of weight to investigate whether objective or subjective measures of weight status (i.e., perceived weight, experiences with weight discrimination, and weight bias internalization), or a combination thereof, provide more robust predictors than BMI.

Our study aligns with scholarly insights showing that people believe weight is controllable, irrespective of their BMI. We extend prior research by showing that weight controllability beliefs are associated with negative self-views and defensive avoidance in people with a high BMI, but not in people with a low BMI. Our findings open interesting routes for future research into the consequences of weight moralization and underscore the relevance of nuanced communication about weight controllability, as well as multidisciplinary collaboration about the self-views and inclusion in society of people with overweight and obesity.

## Data Availability Statement

The raw data supporting the conclusions of this article will be made available by the authors, without undue reservation, to any qualified researcher.

## Ethics Statement

The studies involving human participants were reviewed and approved by the Ethical Commission of the Behavioral Research Lab of the Faculty of Economics and Business (University of Groningen). The patients/participants provided their written informed consent to participate in this study.

## Author Contributions

ST contributed to the conception and design of the study, and wrote the first draft of the manuscript. NG and SF wrote sections of the manuscript. All authors contributed to the final manuscript, and read and approved the submitted version.

## Conflict of Interest

The authors declare that the research was conducted in the absence of any commercial or financial relationships that could be construed as a potential conflict of interest.

## Footnotes

1 We would like to say a word about the terminology we use throughout this manuscript. Terminology is a very important topic and one that we as researchers feel strongly about. We are using “people living with obesity” which is in alignment with the stance of many national obesity charities across the world that represent people living with obesity, is the directed language to be used in many national guidelines relating to obesity, and is in line with the very recent joint international consensus statement (Rubino et al., 2020).

2 Note that this study uses the same dataset collected for Täuber et al. (2018). The data exclusion procedures exactly replicate those reported in Täuber et al. (2018). The complete questionnaire contained questions about the public’s view of weight in terms of morality, questions about shame and rejection (adapted from Gausel et al., 2012, 2016; and inspired by Gausel and Leach, 2011), weight bias internalization (WBIS-M; Pearl and Puhl, 2014), and measures of motivation for dieting (General Motivation Scale, GMS; Pelletier et al., 2004) and exercising (Behavioral Regulation in Exercise Questionnaire, BREQ-3; Markland and Tobin, 2004). Note that the measurements which are not reported in the current paper are reported in Täuber et al. (2018).

3 Data was collected through a third party that performs attention checks in the usual manner, and substitutes respondents who fail the attention check until the agreed upon number of participants is achieved. Further, we performed checks regarding how long respondents took to finish the study (*M* = 12.24 min, *SD* = 7.15 min). Results remained unchanged when we excluded respondents who took more than two standard deviations longer than average (more than or equal to 14.29 min) and one standard deviation less than average (less than 5.10 min). The reported results hence pertain to the complete sample.

4 Not controlling for age and sex did not change the results.
