# Supplementary material for: Exploring Responses to Body Weight Criticism: Defensive Avoidance When Weight Is Seen as Controllable
Source: Front Psychol. 2020 Dec 7;11:598109. doi: 10.3389/fpsyg.2020.598109 (PMC7758617; doi:10.3389/fpsyg.2020.598109)
Supplement: Supplementary file 1 [file Presentation_1.pdf]

This is the supplementary material for Täuber S, Flint SW and Gausel N (2020) Exploring Responses to Body Weight Criticism: Defensive Avoidance When Weight Is Seen as Controllable. Front. Psychol. 11:598109. doi: 10.3389/fpsyg.2020.598109

Appendix

Overview over items measured

Weight Controllability Beliefs: Beliefs About Obese Persons Scale (Allison et al., 1991)

(from 1 = strongly disagree to 7= strongly agree)

- Obesity often occurs when eating is used as a form of compensation for lack of love or attention.
- In many cases, obesity is the results of a biological disorder. (reverse)
- Obesity is usually caused by overeating.
- Most obese people cause their problem by not getting enough exercise.
- Most obese people eat more than non-obese people.
- The majority of obese people have poor eating habits that lead to their obesity.
- Obesity is rarely caused by a lack of willpower. (reverse)
- People can be addicted to food, just as others are addicted to drugs, and these people usually become obese.

Defensive Avoidance: Body Image Shame Scale (BISS; Duarte, Pinto-Gouveia, Ferreira, & Batista, 2014)

(1= never, 2 = rarely, 3 = sometimes, 4 = frequently, 5 = almost always)

- I avoid wearing tight clothes that reveal my body shape.
- I avoid social situations (e.g., going out, parties) because of my physical appearance.
- It bothers me to see my body undressed.
- When I see my body in the mirror, I feel I am a defective person.
- I choose clothes that hide parts of my body that I consider ugly or disproportional.
- The relationship I have with my body prevents me from having an intimate relationship with someone.
- I pay close attention to the movements and posture of my body to hide parts that I do not like.
- I feel bad about myself when I use clothes that reveal my body shape.
- I avoid moving my body (for example, dancing) in public places because I feel I am exposing my physical appearance to the criticism of others.
- I feel uncomfortable in social situations because I feel that people may criticize me because of my body shape.
- There are parts of my body that I prefer to hide.
- My physical appearance makes me feel inferior in relation to others.

- I do not like to exercise in front of others because I am afraid of how they might evaluate me.
- The relationship I have with my physical appearance makes it difficult for me to feel comfortable in social situations.
- Are there settings that you tend to avoid because of your weight or because of what other people might think about you?

Self-to-self relating (Forms of Self-Criticising/Attacking & Self-Reassuring Scale-Short Form) (Sommers-Spijkerman et al., 2017)

(1 = not at all like me, 2 = a little bit like me, 3 = moderately like me, 4 = quite a bit like me, 5 = extremely like me)

Reassured Self

- I am able to remind myself of positive things about myself.
- I find it easy to forgive myself.
- I still like being me.
- I can still feel lovable and acceptable.
- I encourage myself for the future.

Inadequate Self

- I find it difficult to control my anger and frustration at myself.
- There is a part of me that feels I am not good enough.
- I remember and dwell on my failings.
- I think I deserve my self-criticism.
- There is a part of me that wants to get rid of the bits I don’t like.

Hated Self

- I have become so angry with myself that I want to hurt or injure myself.
- I have a sense of disgust with myself.
- I stop caring about myself.
- I call myself names
